# Survival benefit of primary and metastatic tumor resection for colon cancer with liver metastases: A population based, propensity score-matched study

**DOI:** 10.3389/fsurg.2022.959826

**Published:** 2022-08-30

**Authors:** Yunfeng Qiao, Yunfeng Qiao, Huijun Li, Jinge Fu, Shuping You

**Affiliations:** ^1^ First Clinical Medical College, Shandong University of Traditional Chinese Medicine, Jinan, China; ^2^ Cancer Center, Faculty of Health Sciences, University of Macau, Macau, China; ^3^ Department of Imaging Medicine, Taikang Tongji (Wuhan) Hospital, Wuhan, China; ^4^ Department of Anorectal Surgery, Weishi Central Hospital, Kaifeng, China; ^5^ Department of Anus and Bowel Surgery, Jingmen No. 2 People's Hospital, Jingmen, China

**Keywords:** resection, colon, metastases, SEER, liver

## Abstract

**Background:**

Colon cancer remains one of the most common malignancies and we aimed to evaluate whether surgery has an effect on the survival of metastatic colon patients.

**Methods:**

We analyzed 7,583 metastatic colon patients from the Surveillance, Epidemiology, between January 2010 and December 2015. Using Cox proportional hazards models and Kaplan-Meier curves, the overall survival rate (OS) and cancer-specific survival rate and End Results (SEER) registry (CSS) months (m) were evaluated with corresponding 95% confidence intervals (95% CIs). Propensity score matching (PSM) was performed to adjust for potential baseline confounding of all comparison groups.

**Results:**

In general, receiving both primary and metastatic tumor resection (PMTR) remarkably improved OS and CSS compared with only primary tumor resection (PTR) after PS matching (PSM) (*P* < 0.05), with a significantly improved OS (HR = 0.74, 95%CI = 0.69–0.80) and CSS (HR = 0.71, 95%CI = 0.66–0.76) in all stage M1 colon patients. The stratification analysis indicated a significant difference between OS and CSS in M1a and M1b stages. After PSM, PMTR was found to be associated with remarkably improved OS and CSS for patients with liver metastases but not associated with OS and CSS of patients with lung metastases in both M1a and M1b stage.

**Conclusions:**

The results from this large SEER cohort supported PMTR might improve the survival of colon patients with liver metastases on the basis of chemotherapy.

## Introduction

Colorectal cancer (CRC) remains the third most common malignancy diagnosed and the second leading cause of cancer-related deaths globally, with an estimated 1,880,725 new cases and 915,880 deaths worldwide in 2020 (1). Unfortunately, 22% of those were often diagnosed at the metastatic stage American Joint Committee on Cancer (AJCC) stage IV (2). Although a small proportion of metastatic patients might be cured with multimodality treatment, most patients are incurable. Thus, there is a need to improve the survival of metastatic colon cancer patients.

For patients with metastatic colon cancer, palliative colectomy remains important in relieving symptoms, especially in emergencies including severe bleeding, bowel obstruction, or perforation (3). Over the past decade, some studies have demonstrated that primary tumor resection (PTR), despite the presence of unresectable metastatic disease, may improve overall survival (4–6). Recently, it has also been shown that surgery to remove the primary tumor and oligo-metastatic lesions may improve the overall survival (OS) of metastatic CRC (7). However, Luo *et al.* divided the surgery into PTR and metastatic tumor resection respectively, and it remains unclear regarding the role of both primary and metastatic tumor resection (PMTR)*.* Also, this study is lack of the information about M1a stage and M1b stage. Despite a number of studies (4–7) showing improved survival among CRC patients with metastases, the long-term benefit of PMTR has not been clearly defined.

The present study was designed to evaluate the survival benefit of PMTR for patients with M1 colon cancer. In the study, we analyzed a large retrospective cohort of patients with metastases from the SEER registry, through conventional and PSM approaches.

## Methods

### Study population and data sources

**Figure 1:**
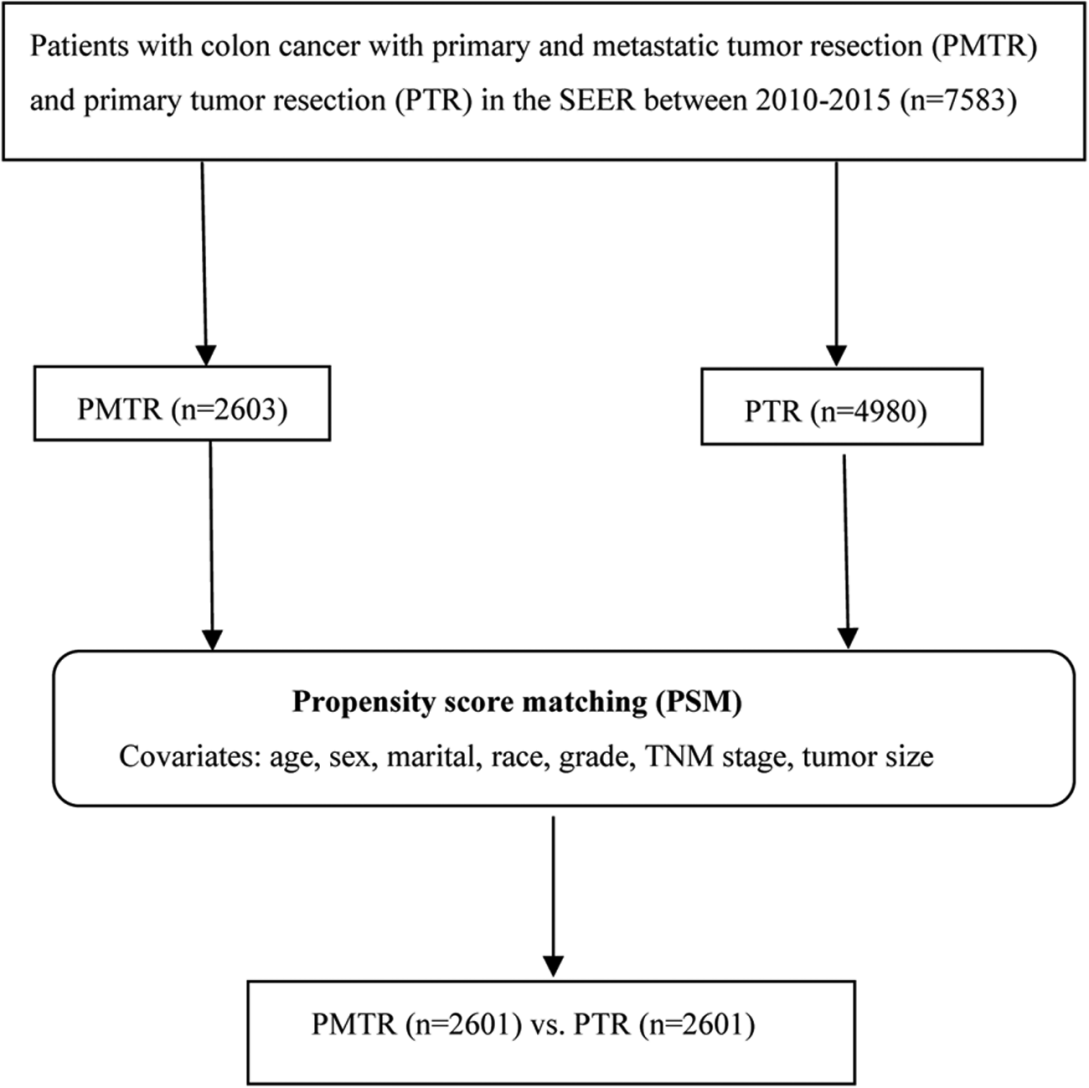
The flowchart of study population selection.

Approximately 34.6% of the US population is covered by the SEER database, which records some clinical parameters and basic demographic information (8). Eligible participants diagnosed as colon cancer with a pathologic report were identified from SEER database between January 2010 and December 2015. Patients were selected using SEER*stat software (version 8.3.4). Patients with the American Joint Committee on Cancer (AJCC) 7th edition stage M1, 80 ≥ age ≥ 18, one primary only, chemotherapy performed, without radiation, more than 3 months survival, active follow up, and had complete data of age, marital, race, sex, grade, TNM stage, tumor size, metastatic sites of lung, liver, bone, brain at diagnose, surgery information were included in our study. Primary tumor resection was defined as surgery performed in the primary site, including total or near-total colectomy and colectomy with a resection in continuity with the resection of other organs (SEER RX Summ–Surg Prim Site (1998+), codes 30, 32, 40, 41, 50, 51, 60, 61, 70, 80 and 90). As a result, a total of 7,583 patients were recruited in this study, including those who received PMTR (cases, *n* = 2,603), and those who received PTR (controls, *n* = 4,980) (Figure 1).

### Propensity score matching (PSM)

Using propensity 1:1 nearest neighbor matching with a 0.02 caliper width, PSM is a tool employed to reduce possible selection bias in non-randomized studies (9). The standardized deviation was performed to check the covariate balance. Variables which remained significant were entered into multivariate logistic regression model; variables left significant in the final model were then analyzed using PSM. Selected covariates were age, sex, race, grade, TNM stage, marital status, and tumor size.

### Statistical analysis

Kaplan-Meier analysis was performed in analyzing cancer-specific survival (CSS) and OS. The distribution of demographic characteristics was analyzed with Chi-squared tests. Univariate and multivariate Cox proportional hazardous model was employed to estimate the hazard ratio (HR) and 95% confidence interval (CI).

In the univariate analysis, variables found to be significant were included in the multivariate analysis, including age (≤60 years old, >60 years old), grade (grade1–2, grade3–4, unknown), marital status (married, unmarried), race (white, black, others), tumor size (≤5 cm, >5 cm), TNM stage, surgery. All *P*-values <0.05 were deemed statistically significant (2-sided). All statistical analyses were performed using SPSS 23.0 (IBM Corp, Armonk, NY, USA).

## Results

The distributions of the characteristics are presented in Table 1 for the study groups that were categorized by receiving PMTR or PTR. Before PSM, the distributions of most demographic factors and clinical factors were in general significantly different between cases and controls. Compared with controls, cases were more likely to be younger, female, smaller tumor size, with well-defined N stage and M stage. In this study, 2,601 pairs were matched using PSM. The distributions of most demographic and clinical factors were well balanced between the cases and controls after PSM.

**Table 1:** Baseline characteristics of study groups before and after matching, SEER 2010–2015.

Characteristic	Before matching			After matching		
	PMTR (*n *= 2603)	PTR (*n *= 4980)	*P value* ^a^	PMTR (*n *= 2601)	PTR (*n *= 2601)	*P value* ^a^
Age [Years, mean (SD)]	56.5 (11.5)	58.9 (11.4)	<0.001	56.5 (11.5)	57.4 (11.4)	0.009
Sex (Female, %)	1,407 (54.1)	2,284 (45.9)	<0.001	1,405 (54.0)	1,374 (52.8)	0.389
Marital (Married, %)	1,537 (59.0)	2,830 (56.8)	0.063	1,535 (59.0)	1,559 (59.9)	0.498
Race (%)			0.162			<0.001
White	1,957 (75.1)	3,657 (73.4)		1,955 (75.1)	1,924 (74.0)	
Black	426 (16.4)	843 (16.9)		426 (16.4)	354 (13.6)	
Other	220 (8.5)	480 (9.7)		220 (8.5)	323 (12.4)	
Grade (%)			0.065			0.723
Grade1–2	1,708 (65.6)	3,261 (65.5)		1,706 (65.6)	1,689 (64.9)	
Grade3–4	765 (29.4)	1,524 (30.6)		765 (29.4)	789 (30.3)	
Unknown	130 (5.0)	195 (3.9)		130 (5.0)	123 (4.7)	
Tumor size			<0.001			0.003
≤5 cm	1,308 (50.2)	2,409 (48.4)		1,306 (50.2)	1,241 (47.7)	
>5 cm	1,093 (42.0)	2,292 (46.0)		1,093 (42.0)	1,200 (46.1)	
Unknown	202 (7.8)	279 (5.6)		202 (7.8)	160 (6.2)	
T (%)			0.092			0.836
T1–2	110 (4.2)	172 (3.5)		108 (4.2)	111 (4.3)	
T3–4	2,493 (95.8)	4,808 (96.5)		2,493 (95.8)	2,490 (95.7)	
N (%)			<0.001			0.412
N0	555 (21.3)	828 (16.6)		553 (21.3)	529 (20.3)	
N1–2	2,048 (78.7)	4,152 (83.4)		2,048 (78.7)	2,072 (79.7)	
M (%)			0.043			0.663
M1a	1,488 (57.2)	2,830 (56.8)		1,486 (57.1)	1,499 (57.6)	
M1b	1,043 (40.0)	1,957 (39.3)		1,043 (40.1)	1,021 (39.3)	
M1nos	72 (2.8)	193 (3.9)		72 (2.8)	81 (3.1)	
Bone metastasis (Yes, %)	17 (0.7)	99 (2.0)	<0.001	17 (0.7)	53 (2.0)	<0.001
Brain metastasis (Yes, %)	6 (0.2)	9 (0.2)	0.643	6 (0.2)	6 (0.2)	1
Liver metastasis (Yes, %)	1,663 (63.9)	3,510 (70.5)	<0.001	1,661 (63.9)	1,817 (69.9)	<0.001
Lung metastasis (Yes, %)	221 (8.5)	778 (15.6)	<0.001	221 (8.5)	388 (14.9)	<0.001

Abbreviations: n, number; PSM, propensity score matching; PTR, only primary tumor resection; PMTR, both primary and metastatic tumor resection.

Entered in PSM were variables including age, sex, marital status, race, grade, TNM stage, and tumor size.

^a^ Derived from ANOVA for continuous variables and X² test for categorical variables.

In the univariate and multivariate analyses of both OS and CSS, all the baseline characteristics and variable selections were included. Table 2 shows the independent risk factors for survival of metastatic colon cancer patients. As expected, older age, black people, higher histology grade, larger tumor size and poorer TNM-stages were related with remarkably worse OS and CSS. It was found that chemotherapy and PMTR were strongly linked to a better survival. Compared with PTR, PMTR showed statistically better survival with a HR (HR_OS _= 0.74, 95% CI, 0.69–0.80, *P < *0.001; HR_CSS _= 0.71, 95% CI, 0.66–0.76, *P < *0.001).

**Table 2:** Univariate and multivariate analyses of OS and CSS to PMTR and PTR groups after matching, SEER 2010–2015.

Charactrastic	Univariate				Multivariate			
	OS		CSS		OS		CSS	
	HR (95% CI)	*P*	HR (95% CI)	*P*	HR (95% CI)	*P*	HR (95% CI)	*P*
Age (≤60 as ref.)								
>60	1.21 (1.12–1.29)	<0.001	1.16 (1.08–1.25)	<0.001	1.20 (1.12–1.29)	<0.001	1.15 (1.06–1.24)	<0.001
Sex (female as ref.)								
Male	1.03 (0.96–1.11)	0.352	1.03 (0.95–1.11)	0.479	1.09 (1.01–1.17)	0.021	1.08 (0.99–1.16)	0.063
Marital (unmarried as ref.)								
Married	0.85 (0.79–0.92)	<0.001	0.88 (0.81–0.95)	0.001	0.85 (0.79–0.92)	<0.001	0.88 (0.81–0.95)	0.001
Race (white as ref.)								
Black	1.14 (1.04–1.25)	0.007	1.14 (1.03–1.27)	0.012	1.22 (1.11–1.34)	<0.001	1.23 (1.11–1.37)	<0.001
Other	0.94 (0.84–1.06)	0.326	0.95 (0.83–1.08)	0.406	0.92 (0.82–1.04)	0.184	0.92 (0.81–1.05)	0.216
Grade (1–2 as ref.)								
Grade3–4	1.81 (1.68–1.95)	<0.001	1.81 (1.67–1.96)	<0.001	1.64 (1.52–1.77)	<0.001	1.64 (1.51–1.78)	<0.001
Unknown	1.09 (0.92–1.29)	0.324	1.02 (0.84–1.23)	0.858	1.17 (0.98–1.39)	0.086	1.10 (0.91–1.34)	0.322
Tumor size (≤5 cm as ref.)								
>5 cm	1.21 (1.13–1.30)	<0.001	1.21 (1.12–1.31)	<0.001	1.16 (1.08–1.25)	<0.001	1.16 (1.07–1.25)	<0.001
Unknown	0.85 (0.73–0.99)	0.034	0.80 (0.67–0.94)	0.007	0.97 (0.83–1.14)	0.729	0.93 (0.78–1.10)	0.378
T (T1–2 as ref.)								
T3–4	1.60 (1.30–1.96)	<0.001	1.59 (1.27–1.98)	<0.001	1.26 (1.03–1.56)	0.028	1.24 (0.99–1.55)	0.066
N (N0 as ref.)								
N1–2	1.81 (1.65–1.99)	<0.001	1.89 (1.70–2.10)	<0.001	1.61 (1.46–1.78)	<0.001	1.67 (1.50–1.86)	<0.001
M (M1a as ref.)								
M1b	1.70 (1.58–1.82)	<0.001	1.69 (1.57–1.83)	<0.001	1.56 (1.45–1.68)	<0.001	1.56 (1.44–1.68)	<0.001
M1nos	1.51 (1.23–1.84)	<0.001	1.44 (1.16–1.80)	0.001	1.59 (1.30–1.94)	<0.001	1.52 (1.22–1.89)	<0.001
Surgery (PTR as ref.)								
PMTR	0.74 (0.69–0.79)	<0.001	0.71 (0.65–0.76)	<0.001	0.74 (0.69–0.80)	<0.001	0.71 (0.66–0.76)	<0.001

Abbreviations: OS, overall survival; CSS, cancer-specific survival; HR, hazard ratio; CI, confidence interval; PTR, only primary tumor resection; PMTR, both primary and metastatic tumor resection.

**Figure 2:**
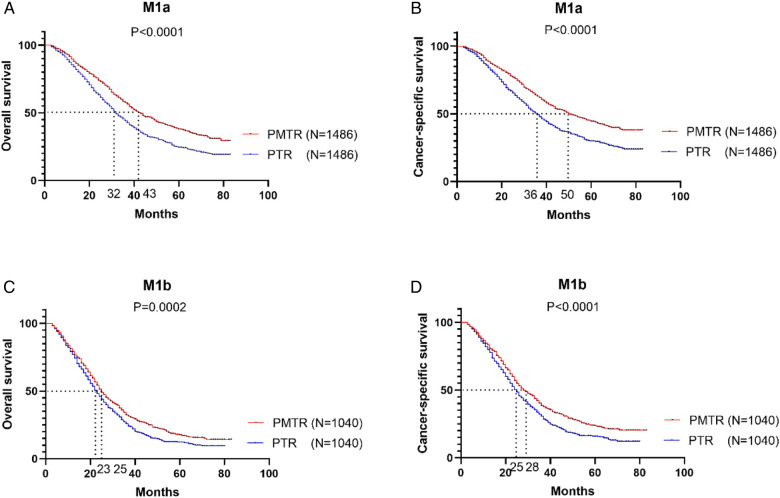
In the stratified analysis between M1a and M1b stage, the Kaplan-Meier survival analysis based on the status of surgical management (both primary and metastatic tumor resection [PMTR], only primary tumor resection [PTR]).

Figure 2 further illustrates the effect of surgery on OS and CSS for patients with stage M1a and M1b after PSM. For patients with M1a (only1 metastatic site), the most striking improvement was observed in PMTR group, with a median OS improved from 32.0 months to 43.0 months (*P *< 0.001) (Figure 2A) and CSS from 36.0 months to 50.0 months (*P *< 0.001) (Figure 2B). In M1b stage (≥2 metastatic sites), the better OS and CSS were observed in PMTR group (median OS = 25.0 months, median CSS = 28.0 months, P < 0.05 compared with PTR groups) (Figures 2C,D).

**Figure 3:**
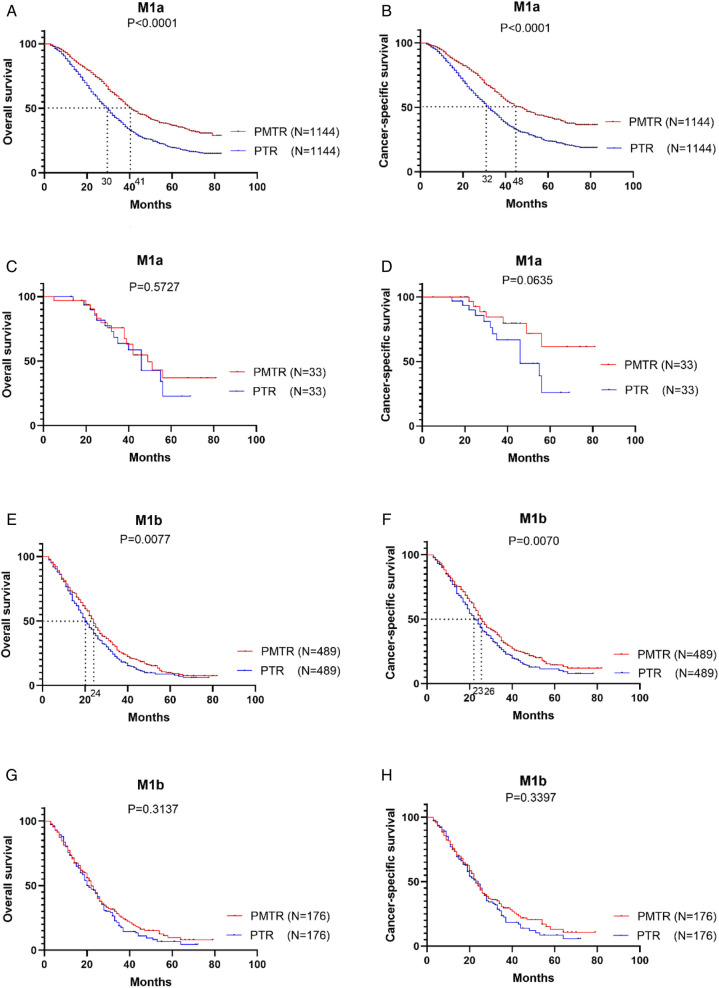
Kaplan–Meier survival cures of the effect of study groups after matching, SEER 2010-2015. (**A**) OS of patients with M1a with only liver metastatic. (**B**) CSS of patients with M1a with only liver metastatic. (**C**) OS of patients with M1a with only lung metastatic. (**D**) CSS of patients with M1a with only lung metastatic. (**E**) OS of patients with M1b with liver metastatic. (**F**) CSS of patients with M1b with liver metastatic. (**G**) OS of patients with M1b with lung metastatic. (**H**) CSS of patients with M1b with lung metastatic.

Furthermore, the prognostic value of the metastatic sites was evaluated (Figure 3). For example, the median OS and CSS for patients with only liver metastasis was improved from 30.0 months to 41.0 months (*Pos *< 0.001) (Figure 3A) and from 32.0 months to 48.0 months (*Pcss *< 0.001) (Figure 3B). However, the sample size of patients with brain, or bone metastasis only was too small to analyze. Furthermore, improved survivals were also displayed in patients with metastasis ≥2 sites. Receiving PMTR could bring a median OS approaching 24.0 months and a median CSS about 26.0 months for patients with liver metastasis, whereas only 20.0 months of OS and 23.0 months of CSS for those received PTR (Figures 3E,F). However, there was no association between PMTR and survival benefit in patients with lung metastases in stage M1a and M1b (Figures 3C,D,G,H).

## Discussion

To our knowledge, the present study is the first population-based analysis to use PSM to assess the role of PMTR in treating metastatic colon cancer. In this study, we found that PMTR, in general, could bring a survival benefit for metastatic colon cancer in both multivariate regression as well as PSM analyses. The beneficial effect of the survival observed from the large cohort of SEER patients highlighted the importance of PMTR in the management of M1 stage colon cancer.

The 5-year survival rate is only approximately 8%–20% for patients with metastatic colon cancer (10–12). For metastatic colon cancer, the effect of palliative colectomy remains debatable. In the past, palliative colectomy was viewed as valueless in treating metastatic disease due to its 20%–30% postoperative morbidity risk and 1%–6% perioperative mortality risk (13, 14). Another study also discovered that palliative resection couldn't improve the survival rate derived from the NCI/SEER database (15). On the other hand, several studies showed that metastatic colon cancer patients who underwent surgery to remove the colon primary lesion might prolong survival than non-operated patients (4–6). By analyzing SEER database, Tarantino *et al.* reported that palliative surgical resection of primary tumor was significantly associated with greater survival rates for patients with unresectable stage IV CRC (HR 0.40, 95% CI 0.39–0.42; *P *< 0.001) (4). Rooijen *et al.* (6) performed a secondary analysis of 8 randomized controlled trials in the ARCAD (Aide et Recherche en Cancérologie Digestive) database. This study found that median OS (16.4 m) for unresected patients was significantly worse than that for the metachronous group (22.4 m; HR 1.81, 95% CI 1.58–2.07) and synchronous resected group (22.2 m; HR 1.60, 95% CI 1.43–1.78). In addition, the unresected group had a significantly worse median progression-free survival than the metachronous (HR 1.47, 95% CI 1.30–1.66) group and synchronous resected (HR 1.31, 95% CI 1.19–1.44) group. Furthermore, some literature reviews and observed analyses of clinical trials have demonstrated that PTR may prolong survival in synchronous mCRC (16–18). Thus, surgical intervention may be an option to extend the survival time in stage IV colon cancer.

The current study showed that PMTR improved the median OS from 32.0 months to 43.0 months and median CSS from 36.0 months to 50.0 months for M1a (namely, oligo-metastatic) colon cancer after PSM. Intriguingly, we found that PMTR for patients with metastatic of two or more organs could still bring a 2 months or higher improvement compared with PTR. Subsequently, a stratified analysis of metastatic sites was performed in M1a and M1b stages. We found that PMTR had better OS and CSS for patients with liver metastases in both M1a and M1b stage. Interestingly, we did not find the survival benefit of PMTR in patients with lung metastases.

Regardless of differences in metastatic sites, resection of the primary tumor and the metastatic site(s) could bring long term survival. Miyoshi *et al.* (19) have reported an observational study in 103 patients with oligo-metastatic disease who underwent PMTR. The postoperative 5-year survival of patients with only liver metastasis (M1a stage) or only lung metastasis (M1a stage) was 43.7 or 90.0%, respectively. Nevertheless, the median OS was 20.7 months for patients with synchronous lung and liver metastases (M1b). Reddy *et al.* (20) reported that simultaneous resection of liver metastasis and colorectal cancer (PMTR) shortened overall hospitalization without compromising safety. Furthermore, Lambert *et al.* (21) discovered that there was no significant difference between survival rates for patients who underwent synchronous and metachronous resections, and that secondary metastases seldom occurred after primary tumor was removed. On the contrary, a study showed increased mortality when synchronous resection of the primary with major hepatectomy (PMTR), with surgical mortality rate of up to 17% (22). However, for carefully selected patients with oligo-metastatic disease, palliative colectomy and surgery of metastases could bring long term survival (23–25).

Our study showed that patients with lung metastases had worse OS and CSS than those with liver metastases. In fact, the survival differences between the two groups may be explained by genetic variations. As reported by Tie et al., lung metastases have a higher frequency of KRAS mutations than liver metastases (26). This phenomenon suggests a KRAS-linked mechanism that promotes colon tumour cell targeting to the lungs (27). Correspondingly, KRAS mutation was associated with lung relapse but not liver relapse in patients from the VICTOR trial (28). Thus, lung metastases are associated with poor prognoses in colon cancer.

When looking at patients with lung metastases who underwent surgery (either PTR alone or PMTR), no difference was found in M1a and M1b stage. As an alternative to lung resection, ablative techniques are often offered to patients with incurable lung metastases (29). Due to its minimally invasive nature, ablative techniques may offer similar survival benefits but less surgical morbidity. Thus, the role of lung resection still remains unclear and hardly generalizable. Based on the results, patients with liver metastases may prolong survival from aggressive treatment such as PMTR, or PTR, while those with lung metastases may only benefit from PTR, and PMTR may not be necessary.

We acknowledge that this study has several limitations. To begin with, like any observational study, bias is always a concern. Using the PSM analyses, we could potentially eliminate the bias from the unbalanced distribution of measured factors. Nevertheless, bias from unmeasured covariates shall be inevitable. Unlike randomized controlled trials, data from SEER registries are generally of high integrity and representative of actual patient populations. Though our findings might apply to real-world patients, we still acknowledge that chemotherapy, surgical methods, and patient status may contribute to study bias.

Besides, the SEER database doesn't offer any information on risk factors of colon cancer, performance status, comorbidities, the sequence of PMTR (metachronous or synchronous), and other metastatic organs, which may have an impact on the survival. A representative database of countries was used to select study participants, so potential selection bias was reduced. Multivariable analyses and PSM analyses were conducted, and OS and CSS results didn't change significantly and therefore appeared valid and stable. Furthermore, the randomized multicenter clinical trials are urgently needed to explore the effect of surgical intervention for metastatic colon adenocarcinoma, and further, to clarify what kind of surgical intervention is most profitable.

## Conclusion

This study from the large SEER database suggests patients with stage M1 colon cancer might benefit from surgical intervention according to the modalities of surgery, even for extensive metastatic patients. Patients with liver metastases may prolong survival from aggressive treatment such as PMTR, or PTR, while those lung metastases may only benefit from PTR, and PMTR may not be necessary.

## Data Availability

The datasets presented in this study can be found in online repositories. The names of the repository/repositories and accession number(s) can be found below: **SEER**.
